# Development and validation of a nomogram for preoperative prediction of cervical lymph node involvement in thyroid microcarcinoma

**DOI:** 10.18632/aging.102915

**Published:** 2020-03-13

**Authors:** Si Lu, Rongjie Zhao, Yeqin Ni, Jinwang Ding, Fuqiang Qiu, You Peng, Gang Pan, Fan Wu, Yu Zhang, Jingjing Shi, Tianhan Zhou, Dingcun Luo

**Affiliations:** 1 Zhejiang Chinese Medical University Affiliated Hangzhou First Hospital, Hangzhou First People’s Hospital, Hangzhou 310006, Zhejiang, China; 2 Department of Medical Oncology, Sir Run Run Shaw Hospital, College of Medicine, Zhejiang University, Hangzhou 310000, Zhejiang, China; 3 Department of Oncological Surgery, Affiliated Hangzhou First People’s Hospital, Zhejiang University School of Medicine, Hangzhou 310006, Zhejiang, China; 4 Department of Ultrasound, The Second Affiliated Hospital of Zhejiang University School of Medicine, Hangzhou 310058, Zhejiang, China

**Keywords:** papillary thyroid microcarcinoma, cervical regional lymph node involvement, SEER, model, nomogram

## Abstract

Cervical regional lymph node involvement (CRLNI) is common in papillary thyroid microcarcinoma (PTMC), but the way to deal with cervical lymph node involvement of clinically negative PTMC is controversial. We studied data of patients histologically confirmed PTMC in the Surveillance, Epidemiology, and End Results (SEER) Program and Department of Surgical Oncology in Hangzhou First People’s Hospital (China). We screened 6 variables of demographic and clinicopathological characteristics as potential predictors and further constructed a lymph node involvement model based on the independent predictors including age, race, sex, extension, multifocality and tumor size. The model was validated by both the internal and the external testing sets, and the visual expression of the model was displayed by a nomogram. As a result, the C-index of this predictive model in the training set was 0.766, and the internal and external testing sets through cross-validation were 0.753 and 0.668, respectively. The area under the receiver operating characteristic curve (AUC) was 0.766 for the training set. We also performed a Decision Curve Analysis (DCA), which showed that predicting the cervical lymph node involvement risk applying this nomogram would be better than having all patients or none patients use this nomogram.

## INTRODUCTION

Papillary thyroid carcinoma (PTC) is the most common malignancy in thyroid cancer. In the past decades, the incidence of PTC has increased rapidly, with a range from 2.9 to 3.2-fold increase [1, 2]. PTMC accounts for 39%-50% of PTC, which is defined as a tumor lesion less than 10 mm [3]. Although PTMC presents an indolent course, with 10-year disease-specific survival rates more than 90% [4], the ratio of CRLNI may occur in 24%-63% of patients at the time of presentation, which has been considered a risk factor for recurrence and distant metastases [5, 6]. Nowadays, thyroidectomy combines with therapeutic lymph node dissection has become a common initial surgical strategy for PTMC patients with clinical lymph nodes positive(cN1). However, the significance of prophylactic central neck dissection (pCND) in clinical lymph nodes negative(cN0) PTMC patients remains controversial. The guidelines from some countries underline positively the necessity of pCND for cN0 PTMC patients, such as China and Japan, for previous studies had found that a large number of pathological lymph nodes positive (pN1) patients were detected after lymph node dissection in PTMC patients with cN0 [7, 8]. Nevertheless, taking the 2015 American Thyroid Association (ATA) guideline as an example, it suggests that pCND is not routinely performed on non-invasive cN0 PTMC. Accordingly, the lack of consensus and standard criterion may leave the surgeon uncertain on how to select the best treatment for patients individually and more evidence is needed. At present, a lot of studies have been done on the risk factors affecting CRLNI in PTMC, but papers constructing risk models to predict CRLNI are very limited, which may be used to guide clinical decisions in the future.

In our study, we aimed to find a more reasonable alternative to evaluate the patient's condition and provide optimal surgical treatments for PTMC patients with CRLNI. We utilized the data of PTMC patients from the SEER Program as well as our medical center to construct a lymph node involvement model for predicting preoperative CRLNI based on the independent predictors including age, race, sex, extension, multifocality and tumor size using statistical method, which was validated by both the internal and external testing set, and the visual expression of the model was displayed by a nomogram.

## RESULTS

### Demographics and characteristics of patients

A total of 22637 cases with non-metastatic histologically confirmed PTMC from 2010 to 2017 were included in our study, of which 21606 cases were from the SEER Program and 1031 from our medical center. All data were divided into three groups which consisted of the training set (n=15124, 70% of SEER), internal testing set (n=6482, 30% of SEER) and external testing set (our center). Approximately 63% of the patients were <55 years old. The ratio of male to female was about 1:4.7, which is similar to the current studies [9–12]. Approximately 94% of the primary cancer sites were confined within the thyroid capsule. The majority of cases were lymph node negative. The demographics and characteristics of different data sets are summarized in Table 1.

**Table 1:** Differences of demographics and characteristics for M0 patients of PTMC in different data sets.

**Demographics and characteristics**	**Training data (n=15124)**	**Internal testing data (n=6482)**	**External testing data (n=1031)**	**Total (n=22637)**
Age (year)				
<55	9388 (62.07)	4081 (62.96)	807 (78.27)	14276 (63.06)
≥55	5736 (37.93)	2401 (37.04)	224 (21.73)	8361 (36.94)
Race				
Other^1^	1524 (10.08)	655 (10.10)	1031	3210 (14.18)
white	12503 (82.67)	5391 (83.17)	/	17894 (79.05)
black	1097 (7.25)	436 (6.73)	/	1533 (6.77)
Sex				
Male	2666 (17.63)	1145 (17.66)	186 (18.04)	3997 (17.66)
Female	12458 (82.37)	5337 (82.34)	845 (81.96)	18640 (82.34)
Tumor size (mm)	5.51±2.94	5.51±2.97	5.50±2.41	5.51±2.93
Extension				
intrathyroidal extension	14194 (93.85)	6081 (93.81)	967 (93.80)	21242 (93.84)
minimal extension	868 (5.74)	375 (5.79)	52 (5.04)	1295 (5.72)
gross extension	62 (0.41)	26 (0.40)	12 (1.16)	100 (0.44)
Multifocality				
Solitary tumor	9684 (64.03)	4173 (64.38)	803 (77.89)	14660 (64.76)
Multifocal tumor	5440 (35.97)	2309 (35.62)	228 (22.11)	7977 (35.24)
N status				
positive	1812 (11.98)	771 (11.89)	367 (35.60)	2950 (13.03)
negative	13312 (88.02)	5711 (88.11)	664 (64.40)	19687 (86.97)

Notes: All data was presented as the number of cases and composition ratio (%) or mean ± standard deviation. ^1^Including American Indian/AK Native, Asian/Pacific Islander.

Abbreviations: M0 = no distant metastases; PTMC = papillary thyroid microcarcinoma.

### Predictors selection

**Figure 1:**
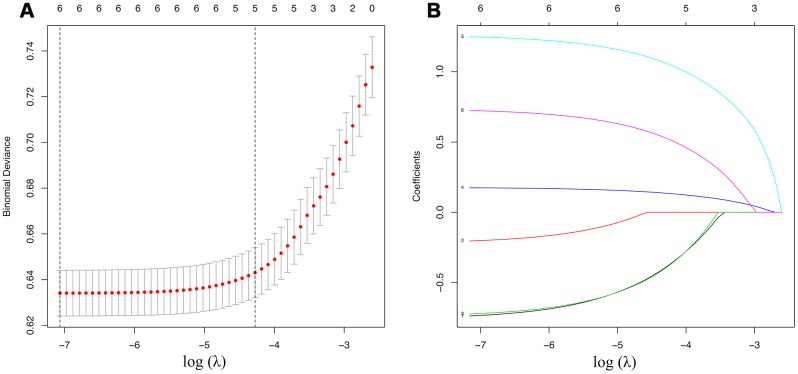
**Demographic and clinicopathological characteristics screening applying the LASSO logistic regression model.** Final predictors include age, race, sex, extension, multifocality, tumor size. (**A**) Suitable parameter (λ) selection in the LASSO model used 5-fold cross-validation via minimum criteria [38–40]. We plotted the partial likelihood deviance (binomial deviance) curve versus log (λ). 2 dotted vertical lines were drawn at the optimal values applying the minimum criteria and the 1 standard error of the minimum criteria (the 1-SE criteria). (**B**) LASSO coefficient profiles of the 6 variables. We produced a coefficient profile plot against the log (λ) sequence. A suitable λ was chosen when log (λ)= -5 and resulted in 6 variables with nonzero coefficients. LASSO=least absolute shrinkage and selection operator, SE=standard error.

A total of 1812 in 15124 cases of training date set had positive cervical regional lymph nodes (12%). All the variables were found to be significantly correlated with cervical regional lymph nodes involvement via univariate analysis (Table 2) including age, race, sex, extension, multifocality, tumor size. To avoid the influence of confounding factors, we performed a LASSO regression analysis to re-evaluate the variables. Finally, we retained 6 variables with nonzero coefficients (Figure 1) as potential predictors of the prediction model. These predictors included age, race, sex, extension, multifocality, tumor size.

**Table 2:** Risk factors of cervical regional lymph node involvement in the training and testing data sets.

**Risk factors**	**SEER database**							**Our medical center**		
	**Training data (n=15124)**			**Internal testing data (n=6482)**				**External testing data (n=1031)**		
	**Node positive**	**Node negative**	**p value**	**Node positive**	**Node negative**	**p value**		**Node positive**	**Node negative**	**p value**
Age (year)			<.001			<.001				0.11
<55	1374(75.83)	8014(60.20)		572(74.19)	3509(61.44)			298(81.20)	509(76.66)	
≥55	438(24.17)	5298(39.80)		199(25.81)	2202(38.56)			69(18.80)	155(23.34)	
Race			<.001			<.001				
Other^1^	233(12.31)	1301(9.77)		99(12.84)	556(9.74)			367	664	
white	1536(84.77)	10967(82.38)		652(84.57)	4739(82.98)			/	/	
black	53(2.92)	1044(7.84)		20(2.59)	416(7.28)			/	/	
Sex			<.001			<.001				<.001
Male	495(27.32)	2171(16.31)		202(26.20)	943(16.51)			91(24.80)	95(14.31)	
Female	1317(72.68)	11141(83.69)		569(73.80)	4768(83.49)			276(75.20)	569(85.69)	
Tumor size (mm)	7.18±2.46	5.29±2.93	<.001	7.07±2.58	5.30±2.95	<.001		6.26±2.37	5.09±2.33	<.001
Extension			<.001			<.001				0.0095
intrathyroidal extension	1428(78.81)	12766(95.90)		603(78.21)	5478(95.92)			333(90.73)	634(95.48)	
minimal extension	353(19.48)	515(3.87)		152(19.71)	223(3.90)			27(7.36)	25(3.77)	
gross extension	31(1.71)	31(0.23)		16(2.08)	10(0.18)			7(1.91)	5(0.75)	
Multifocality			<.001			<.001				0.0001
Solitary tumor	754(41.61)	8930(67.08)		322(41.76)	3851(67.43)			261(71.12)	542(81.63)	
Multifocal tumor	1058(58.39)	4382(32.92)		449(58.23)	1860(32.57)			106(28.88)	122(18.37)	

Notes: All data was presented as the number of cases and composition ratio (%) or mean ± standard deviation. ^1^Including American Indian/AK Native, Asian/Pacific Islander. PTMC = papillary thyroid microcarcinoma; SEER = Surveillance, Epidemiology, and End Results.

### Construction and validation of predictive model

**Figure 2:**
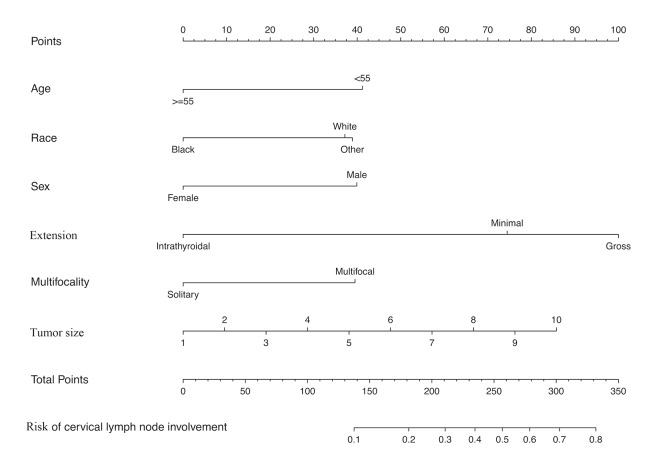
**Nomogram for predicting CRLNI in PTMC patients.** CRLNI=cervical regional lymph node involvement.

To get a more comprehensive view of the relationship between the status of cervical lymph node involvement and these predictors, we further performed a multivariable logistic regression analysis and constructed a predictive model. The results of the logistic regression analysis were given in Table 3 and visualized in the form of a nomogram plot to help practice in the clinic (Figure 2).

**Table 3:** Multivariate logistic regression analysis for cervical regional lymph node involvement in patients with PTMC.

**Predictors**	**β**	**Odds ratio (95% CI)**	**P-value**
Age (vs <55 years)	-0.7596	0.4679(0.4142–0.5275)	<.001
Race (vs Other^1^)			
white	-0.0318	0.9687(0.8245–1.1425)	0.702
black	-0.7168	0.4883(0.3507–0.6692)	<.001
Sex (vs male)	-0.7357	0.4792(0.4239–0.5422)	<.001
Tumor size (mm)	0.1756	1.1920(1.1684–1.2164)	<.001
Extension (vs intrathyroidal extension)			
minimal extension	1.3721	3.9436(3.3669–4.6155)	<.001
gross extension	1.8432	6.3166(3.7236–10.7269)	<.001
Multifocality (vs Solitary tumor)	0.7279	2.0706(1.8616–2.3036)	<.001

Notes: ^1^Including American Indian/AK Native, Asian/Pacific Islander.

Abbreviations: PTMC = papillary thyroid microcarcinoma; OR, odds ratio; CI, confidence interval.

**Figure 3:**
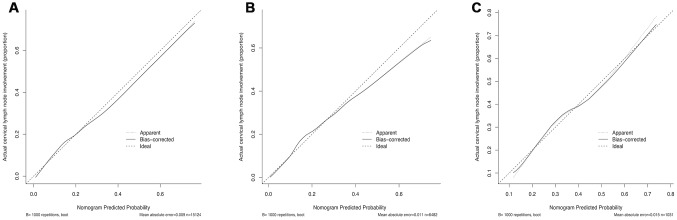
**Calibration curves of the nomogram for predicting CRLNI in PTMC patients.** (**A**) Calibration curve of the nomogram for training set. (**B**) Calibration curve of the nomogram for internal testing set. (**C**) Calibration curve of the nomogram for external testing set. The x-axis represents the predicted CRLNI. The y-axis represents the actual CRLNI. The diagonal dotted line stands for a perfect prediction using an ideal model. We drew the solid line to represent the performance of the nomogram, of which the closer fit to the diagonal dotted line represents the better prediction of the nomogram.

**Figure 4:**
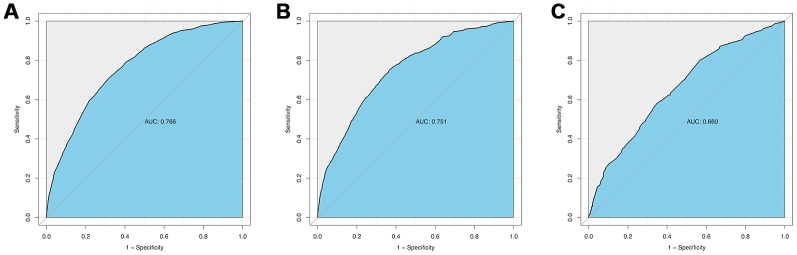
**ROC curve analysis to predict CRLNI in PTMC patients.** (**A**) ROC curve for the training set. (**B**) ROC curve for the internal testing set. (**C**) ROC curve for the external testing set. AUC=area under ROC curve; ROC= receiver operating characteristic.

**Figure 5:**
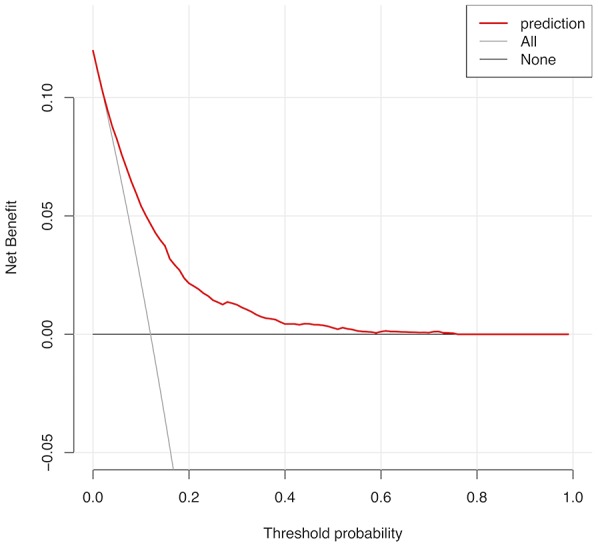
**Decision curve analysis for CRLNI in PTMC patients.** The y-axis represents the net benefit. The red line represents the nomogram of CRLNI. The grey line displays the assumption that all patients have CRLNI. The black line represents the assumption that no patients have CRLNI. The decision curve showed that predicting the CRLNI risk applying this nomogram would be better than having all patients or none patients treated by this nomogram with a range of the threshold probability between >1% and <75.

The calibration curve of the cervical regional lymph node involvement risk nomogram in PTMC suggested great agreement in both training data set and testing data sets including internal and external testing sets, and all the Mean absolute error ≤ 0.015 (Figure 3). The C-index of this predictive model in the training set was 0.766 (95%CI: 0.755–0.777), and the internal and external testing sets through cross-validation were 0.753 and 0.668, respectively. AUC was 0.766 for the training set, and the internal and external testing sets were 0.751 and 0.660 (Figure 4), which suggested the good prediction capability of this model. We also performed a Decision Curve Analysis (DCA), which showed that predicting the cervical regional lymph node involvement risk applying this model would be better than having all patients or none patients treated by this model with a range of the threshold probability between >1% and <75% (Figure 5).

## DISCUSSION

CRLNI is common among PTMC patients, but the way to deal with cervical lymph node involvement of clinically negative PTMC is controversial.

There were 22637 patients with histologically confirmed PTMC from 2010 to 2017 in our study, of which 21606 patients were from the SEER Program from 2010 to 2015 and 1031 patients from our medical center from 2010 to 2017. We focused on the pattern and frequency of lymph node involvement in all patients with PTMC. The prevalence of CRLNI obtained by SEER Program and our medical center was quite different, which was 12.0% (2583 of 21606) and 35.6% (367 of 1031), respectively. One of the causes leading to this large range may be due to the differences in surgical rationales, therapeutic or prophylactic, underlying the use of CND in PTMC. Although PTMC was an indolent tumor, some studies have reported that PTMC patients with CRLNI were more prone to tumor recurrence and implied a worse prognosis [13–15].

In our study, younger age (<55 years), the other race (American Indian/AK Native, Asian/Pacific Islander), male gender, gross extension (nerves, esophagus, larynx, sternocleidomastoid muscle, etc), multifocality and increased tumor size were significantly associated with CRLNI. This was consistent with the present results reported by others in patients with PTMC [5, 12, 16, 17]. Nevertheless, it was not agreed on certain risk factors of CRLNI in PTMC. Siddiqui et al. found Size of tumor and Gender did not correlate with central lymph node involvement while Zheng et al. reported Male gender and increased tumor size (≥5 mm) were related to a significantly higher risk of PTMC central lymph node involvement [11, 12]. Similar positive results have been shown by other researches [18]. In addition, Kim et al. found bilaterality was associated with central lymph node involvement [10]. As for the variable Race, it has been reported that white race was associated with larger tumor size, while tumor enlargement implied a higher risk of CRLNI as mentioned before, indirectly indicating that white race was related to CRLNI, but we found it does not correspond to the results presented by nomogram in this article, which suggested that other race was more likely to cause CRLNI than white race [16]. In line with our results, more studies suggested other race (American Indian/AK Native, Asian/Pacific Islander) was more prone to have lymph node involvement [9]. Besides, since the version of AJCC was updated to the eighth edition, the threshold of age at diagnosis for high-risk of disease-specific mortality has raised from 45 years to 55 years and the superior mediastinum lymph nodes of cervical level VII have been reassigned from N1b (lateral neck) to N1a (central neck) [19]. Based on this modification, data in the current study was divided into groups by 55 years old and status of lymph node involvement was not subdivided into the central neck and lateral neck, as data of SEER database was still accordance with the seventh edition of AJCC.

Few researches were reported on nomogram predicting CRLNI in patients with PTC, especially with PTMC [20–24]. Wang et al. performed a nomogram to predict the risk of level V lymph node involvement [20]. Similarly, Hei et al. performed another nomogram for predicting lateral neck lymph node involvement [25]. Although their purpose was not the same, there existed common deficiencies such as small sample size (1037 and 505 samples) and the absence of external validation. Both models were internally validated through a bootstrapping analysis, which may lead to over-fitting of the model. Moreover, in the study by Kim, their model was validated by both internal and external data sets, but the AUC value of the training set was only 0.721 [22]. Meanwhile, these researches focused on nomograms predicting CRLNI in patients with PTC rather than PTMC. In our study, we analyzed the data based on the SEER database to construct a preoperative model that was validated by internal and external data sets, including data from our medical center. In this model, the AUC value and C-index of the training set were both 0.766 and calibration curve suggested the actual probability of CRLNI corresponded closely with the predicted probability of CRLNI in PTMC patients, which indicated relatively good discrimination ability [26]. Furthermore, the AUC values and C-index were maintained at a good level in both internal and external validation sets.

At present, the significance of pCND in cN0 PTC patients remains controversial, especially in PTMC. On one hand, some countries such as China and Japan underline positively the necessary of the routine pCND for cN0 PTMC [27], which can not only reduce local recurrence rate, but also identify the stage of disease to guide subsequent treatment such as TSH (Thyroid-stimulating hormone) suppression and RAI (Radioactive iodine) treatment [5]. A meta-analysis enrolled in 17 studies observed the locoregional recurrence of PTC patients after surgery, showed that pCND reduced the risk of lymph node recurrence by 34% [28]. Barczyński et al also showed the 10-year local recurrence rates of patients with and without pCND were 5.6% and 13.4%, respectively [29]. On the other hand, the American Thyroid Association (ATA) and British Thyroid Association (BTA) guidelines suggest that pCND is not routinely performed on non-invasive cN0 PTMC [30, 31]. According to the current literature, some studies have reported that there was no significant difference in recurrence rates, regardless of pCND [8, 32]. And pCND might be associated with a higher risk of complication, including hypoparathyroidism and recurrent laryngeal nerve injury. Zhao et al had found that over-treatment increased the incidence of transient and permanent hypoparathyroidism by 2.52 and 1.82, respectively [28]. In addition, ultrasound, the first-line diagnostic approach to assess CRLNI in PTMC patients preoperatively, has poor sensitivity ranges from 20%-50% [33, 34]. As a result, those cN0 PTMC patients could be missed preoperatively.

This study has several limitations. First, the data used to construct the model came from the SEER database, so the types of variables were limited. Imaging features, pathological subtypes and genotypes of fine-needle aspiration (FNA) sample preoperatively might be further added in the future predictive model. Second, the external validation of this model was only performed with single-center data from China. Thus, further evaluation with follow-up data from multicenter and countries is indicated. Finally, our model was only applied to PTMC, not for other pathology types of thyroid cancer.

In summary, we combined clinicopathological data from the SEER database and our medical center to establish an effective nomogram for assessing CRLNI of PTMC. For PTMC patients with a high score on the nomogram, clinicians may consider pCND and strict postoperative evaluation.

## MATERIALS AND METHODS

### Data selection from the SEER

The data we analyzed extracted from two parts, one from the SEER database, and the other from our medical center (Department of Surgical Oncology, Hangzhou First People's Hospital). The SEER program is a population-based cancer registry, which accumulates information on cancer incidence and survival from 17 population-based registries, covering up to 28% of the US population [35]. The data from the SEER program contain no identifiers and are publicly obtained for academic studies. The SEER*Stat software produced by the Surveillance Research Program and National Cancer Institute (version 8.3.5; https://www.seer.cancer.gov/seerstat) is applied to identify the single primary PTMC patients who had undergone surgical treatment. In order to ensure the standardization of information, SEER has used the derived AJCC stage group (7th edition) since 2010, thus only patients diagnosed from 2010 to 2015 with histopathology codes of the International Classification of Diseases for Oncology, 3rd edition (ICD-O-3):8050/3, 8260/3, 8340/3-8344/3 at M0 stage were included in our study. We excluded patients whose clinicopathological profiles were unknown or unclear and pathological type is non-papillary carcinoma (thyroid medullary/anaplastic carcinoma, etc). Nx (lymph nodes cannot be assessed) cases were also not included in this study.

### Data selection from our medical center

At the same time, we collected 1031 patients with PTMC confirmed by operation and pathology from 2010 to 2017 in Hangzhou First People’s Hospital as external testing set for the model, who had no history of other malignant tumors. This project was approved by our hospital ethical committee and adhered to rules of STROCSS criteria [36] with an irreplaceable identifying code (UIN: 3665) from the Research Registry.

### Assessment of clinicopathologic variables

The dichotomous response variable of this model was the status of cervical lymph node involvement, which was divided into N0 and N1 (N1a+N1b). Then we extracted the following variables from the SEER as risk factors of cervical regional lymph node involvement in PTMC: age, race, sex, extension, multifocality, tumor size. Tumor size as a continuous variable was defined as the largest diameter of the primary PTMC. The race was classified as Black, White and other (American Indian/AK Native, Asian/Pacific Islander) provided by the SEER. According to the most recent 8^th^ revision of TNM by the American Joint Committee on Cancer (AJCC), the threshold of age for high-risk of disease-specific mortality was updated form 45 years to 55 years, thus patients in this study were divided into two groups: younger patients (<55 years) and older patients (≥55 years). Multifocality was categorized as a solitary tumor and multifocal tumors. Multifocal tumors were defined as the presence of two or more sites in the thyroid gland, while a solitary tumor represented only one site within the thyroid. The extension of the primary PTMC was stratified into 3 groups: extension was confined within the thyroid capsule, minimal extension (strap muscle) and gross extension (nerves, esophagus, larynx, sternocleidomastoid muscle, etc). The multifocality and extension were both based on the final pathology.

### Statistical analysis

We tried to find out the relationship between characteristics and cervical regional lymph node involvement in patients with PTMC. The Pearson chi-square test and t-test were applied for univariate analysis. Then all data from the SEER database was divided into two groups for cross-validation: 70% for a training data set, and 30% for an internal testing data set. The data from our medical center was used as the external testing data set. We screened out the optimal variables with nonzero coefficients as potential predictors of this prediction model using the least absolute shrinkage and selection operator (LASSO) method [37]. Then multivariable logistic regression analysis was used to construct the predictive model based on the results of LASSO regression and a further nomogram was developed. Odds ratios (ORs) having 95% confidence intervals (CIs) and P-value was calculated. The prediction efficiency of this predictive model was assessed by C-index and AUC as well as calibration curves in both training data set and testing data sets including internal and external sets. DCA curve was also performed to determine the clinic value of the predictive model by quantifying the net benefit at disparate threshold probabilities. All statistical analyses were conducted using R software (version 3.5.1; https://www.r-project.org). Statistical significance was decided by a criterion of two-sided P<0.05.
